# Effect of a home based, low intensity, physical exercise program in older adults dialysis patients: a secondary analysis of the EXCITE trial

**DOI:** 10.1186/s12877-018-0938-5

**Published:** 2018-10-20

**Authors:** Rossella Baggetta, Graziella D’Arrigo, Claudia Torino, Samar Abd ElHafeez, Fabio Manfredini, Francesca Mallamaci, Carmine Zoccali, Giovanni Tripepi, on behalf of the EXCITE Working group, Davide Bolignano, Nicola Lamberti, Silvio Bertoli, Daniele Ciurlino, Lisa Rocca-Rey, Antonio Barillà, Yuri Battaglia, Renato Mario Rapanà, Alessandro Zuccalà, Graziella Bonanno, Pasquale Fatuzzo, Francesco Rapisarda, Stefania Rastelli, Fabrizio Fabrizi, Piergiorgio Messa, Luciano De Paola, Luigi Lombardi, Adamasco Cupisti, Giorgio Fuiano, Gaetano Lucisano, Chiara Summaria, Michele Felisatti, Enrico Pozzato, Anna Maria Malagoni, Pietro Castellino, Filippo Aucella, Pasquale Fabio Provenzano, Luigi Catizone

**Affiliations:** 1 Clinical Epidemiology and Physiopathology of Renal Diseases and Hypertension of Reggio Calabria, National Council of Research, Institute of Clinical Physiology, Reggio Calabria, Italy; 2 Department of Biomedical and Surgical Specialties Sciences, Section of Sport Sciences and Vascular Diseases Center, University of Ferrara, Ferrara, Italy; 3 Epidemiology Department, High Institute of Public Health-Alexandria University, Alexandria, Egypt

**Keywords:** Dialysis patients, Physical exercise, Quality of life

## Abstract

**Background:**

Older adults dialysis patients represent the frailest subgroup of the End Stage Renal Disease (ESRD) population and physical exercise program may mitigate the age-related decline in muscle mass and function.

**Methods:**

Dialysis patients of the EXCITE trial aged > 65 years (*n* = 115, active arm, *n* = 53; control arm, *n* = 62) were submitted in random order to a home based, low intensity physical exercise program. At baseline and 6 months after exercise training 6-min walking distance (6MWD) and 5-time sit-to-stand test (5STS) were performed, and quality of life (QoL) was tested.

**Results:**

The training program improved both the 6MWD (6-months: 327 ± 86 m versus baseline: 294 ± 74 m; *P* < 0.001) and the 5STS time (6-months: 19.8 ± 5.6 s versus baseline: 22.5 ± 5.1 s; P < 0.001) in the exercise group whereas they did not change in the control group (*P* = 0.98 and 0.25, respectively). The between-arms differences (6 months-baseline) in the 6MWD (+ 34.0 m, 95% CI: 14.4 to 53.5 m) and in the 5STS time changes (− 1.9 s, 95% CI: -3.6 to − 0.3 s) were both statistically significant (*P* = 0.001 and *P* = 0.024, respectively). The cognitive function dimension of QoL significantly reduced in the control arm (*P* = 0.04) while it remained unchanged in the active arm (*P* = 0.78) (between groups difference *P* = 0.05). No patient died during the trial and the training program was well tolerated.

**Conclusions:**

This secondary analysis of the EXCITE trial shows that a home-based, exercise program improves physical performance and is well tolerated in elderly ESRD patients.

**Trial registration:**

The trial was registered in ClinicalTrials.Gov (Clinicaltrials.gov identifier: NCT01255969) on December 8, 2010.

**Electronic supplementary material:**

The online version of this article (10.1186/s12877-018-0938-5) contains supplementary material, which is available to authorized users.

## Background

Sedentary lifestyle and reduced physical performance are hallmarks of dialysis patients [1] and both these conditions negatively impact upon general health status and quality of life in this patient-population [2, 3]. NKF-KDOQI Guidelines formally recommend patients on dialysis be encouraged to increase their level of physical activity [4] and a recent randomized, controlled, multicenter, clinical trial (Clinicaltrials.gov identifier: NCT01255969) demonstrated that a personalized, low intensity, home-based, exercise program improved physical performance and had a favourable impact on cognitive function and quality of social interaction (as assessed by KDQOL-SF) in dialysis patients [5]. Furthermore, a corollary analysis of the EXCITE trial showed that a poor physical performance, as assessed by the 6-min walking distance [6], predicted a high risk of mortality, cardiovascular events and hospitalizations in the dialysis population [7].

Patients over 65 years of age constitute the fastest growing segment of ESRD population worldwide [8] and although dialysis treatment increases life expectancy among older patients on chronic dialysis, many uremic patients suffer from distressing physical limitations [9]. Since aging is associated with a decline in muscle mass and performance [10], physical exercise is one of the potential methods to counterbalance age-related worsening in muscle mass and function [11]. Notwithstanding, to date very few studies investigated the effect of physical exercise on clinical outcomes in older patients on dialysis.

With this background in mind we specifically designed a secondary analysis of the Exercise Introduction to Enhance Performance in Dialysis (EXCITE) trial [5] by investigating the efficacy and safety of a low intensity exercise program on physical performance and quality of life in the subgroup of patients of the EXCITE trial aged > 65 years.

## Methods

### Ethical approval and consent to participate

The original protocol of the EXCITE trial was approved by the Ethics Committees of Renal Units (*n* = 9 Italian Centers) participating to the study, and written informed consent was obtained from each participant. The Ethical Committee of the Coordinating Center is that of “Grande Ospedale Metropolitano Bianchi-Melacrino-Morelli” of Reggio Calabria, Italy (Committee’s reference number: CE150157). The trial was registered in ClinicalTrials.Gov (Clinicaltrials.gov identifier: NCT01255969) on December 8, 2010.

### Study design

EXCITE is a centrally randomized (simple randomization), multicenter, open label, controlled, two parallel arms, clinical trial testing the effect of a personalized, home-based, low-intensity program [see: https://www.youtube.com/watch?v=ki8YX_t-0jA, 5] of walking exercise (exercise group) versus usual care (control group, receiving generic advice to maintain an active lifestyle) on physical performance [as assessed by 6-min walking distances (6MWD) and 5-time Sit-To-Stand (5STS) tests] and clinical outcomes (including quality of life) in dialysis patients [6, 12–14]. The walking cadence (steps/minute) to be maintained at home was dictated by an easily available metronome (Seiko DM50; Seiko Ltd., Japan) which was distributed to all participants. The adherence to the prescribed program was assessed by evaluating the residual battery charge in the metronome at the end of the trial. Exclusion criteria were physical or clinical limitations or a high degree of fitness, that is the ability to walk a distance of 550 m in 6 min during the standard walking test (see Ref. [6]). All eligible patients were recruited between November of 2009 and February of 2011. This secondary analysis focuses on the subgroup of patients who took part to the EXCITE trial and aged > 65 years (*n* = 115). The enrolment period ranged from April 2010 to February 2011.

### Laboratory data

Serum cholesterol, triglycerides, hemoglobin, albumin, calcium, phosphate, PTH, glucose, and C-Reactive Protein were measured by standard methods in the routine clinical laboratory.

### Functional capacity tests

The testing sessions were always arranged in a non-dialysis day, 24 h after the dialysis session, either in the morning (between 7 a.m. and 1 p.m.) or in the afternoon (between 2 p.m. and 6 p.m.). Functional capacity testing in both study arms (exercise and control groups) was performed at baseline and after 6 months, using the 6-MWD and the 5-STS. Fatigue during the exercise was assessed by the Borg scale. In the active arm of the trial, the exercise programme was permitted to be done at home or outdoor, dependent on patients’ preferences. Low and high adherence were defined as performance of < 60% and ≥ 60% of the prescribed sessions, respectively. Patients in the control group was given just generic advice to maintain an active lifestyle.

### Quality of life

Quality of life was measured by the KDQOL-SF in the version translated into Italian and specifically validated in a sample of Italian patients with CKD. Whenever needed, the compilation of the replies to the KDQOL-SF was helped by nurses unaware of the treatment allocation of patients. For the purpose of this paper, only changes occurring over a 6-month follow-up period were considered in the data analysis.

### Statistical analyses

Data are expressed as mean and SD (normally distributed data), median and interquartile range (non-normally distributed data), or as percentage frequency (categorical data), and comparisons between groups were made by independent t-test (normally distributed data), Mann–Whitney test (non-normally distributed data), or chi-squared test (categorical data), as appropriate. Within-group comparisons were done by paired t-test (normally distributed data) or Wilcoxon rank test (non-normally distributed data), as appropriate. Between- and within-group differences were expressed as mean changes and 95% confidence intervals (CIs).

An intention-to-treat (ITT) approach was used for the primary study outcomes (the 6MWD and the 5STS). The potential confounding effect of baseline variables which differed (with *P* < 0.10) between the two study arms on the study results was tested by multiple linear regression analysis (for changes in 6MWD and 5STS). Given the fact that this is a secondary analysis of a previous clinical trial, no sample size calculation was reported. The effect of physical exercise on the incidence rate of all-cause and cardiovascular hospitalizations according to study arms was investigated by Cox regression method. In this analysis, data were expressed as hazard ratio (HR), 95% CI and *P* value.

Data analysis was performed using a standard statistical package (SPSS for Windows, version 22; IBM SPSS, Chicago, IL, USA).

## Results

**Fig. 1:**
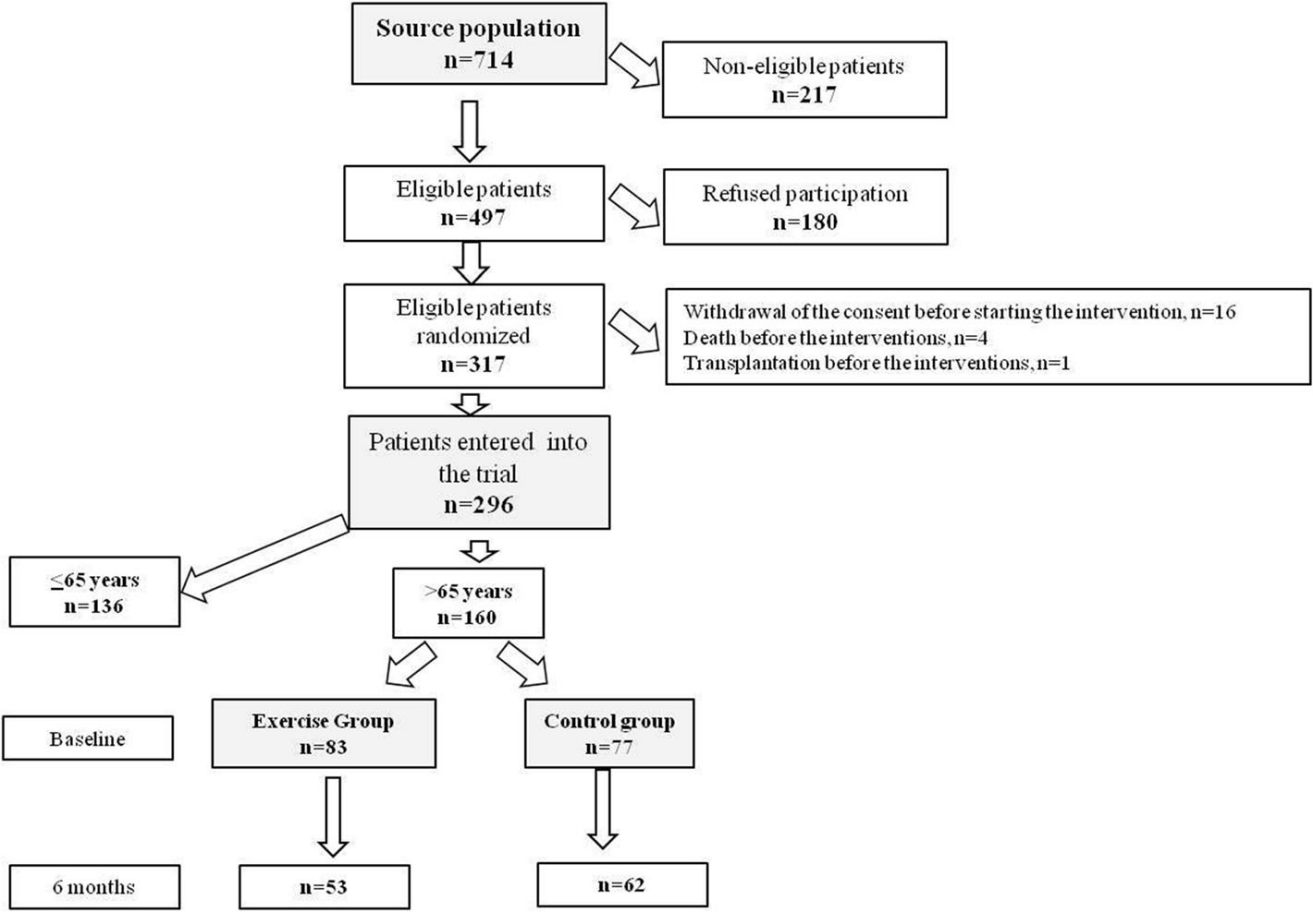
CONSORT diagram describing the flow of patients through the trial

The CONSORT diagram describing the flow of patients through the trial is shown in Fig. 1. The source population was composed by 714 patients and among these 418 patients were excluded for various reasons (Fig. 1). Thus, 296 patients were randomized to walking exercise (*n* = 151) or to usual care/normal physical activity (*n* = 145). As depicted in Figs. 1, 160 patients of 296 (54%) fulfilled the criterion to have an age over 65 years (active arm, *n* = 83; control arm, *n* = 77) and were considered in this secondary analysis. Older adults patients (i.e. patients aged > 65 years) who completed the study at 6 months (active arm, *n* = 53; control arm, *n* = 62) in the two study arms were quite similar as for demographic, clinical, and biochemical data (Table 1) but differed for BMI, systolic BP, hemoglobin, and albumin (Additional file 1: Table S1) which were higher in patients in the active than in those in the control arm. Patients in the active arm had also less frequently stroke/TIA in their clinical history (Table 1). Moreover, age tended to be lower and diastolic BP to be higher (*P* = 0.06 and *P* = 0.07, respectively) in patients allocated to the active arm than in those to the control arm (Table 1). Among patients allocated to the active arm, 26 had low (49%) and 27 high (51%) adherence to the physical exercise program.

**Table 1:** Demographic, clinical and biochemical data of patients that completed the study

	Active arm (*n* = 53)	Control arm (*n* = 62)	P Value
Age, yr	73 ± 5	75 ± 6	0.06
Men, %	64	66	0.82
Hemodialisys/CAPD, n	44/9	55/6	0.25
BMI, Kg/m2	27 ± 4	25 ± 3	0.01
Smokers, %	17	8	0.16
Diabetics, %	17	20	0.75
Myocardial infarction, %	15	19	0.55
Stroke/transient ischemic attack, %	4	16	0.03
Anginal episodes, %	11	16	0.46
Arrhythmia, %	17	13	0.54
Heart failure, %	19	27	0.28
Peripheral vascular disease, %	7	16	0.16
History of neoplasia, %	21	28	0.38
Antihypertensive therapy, %	78	64	0.11
NYHA class, %			0.32
I	40	33	
II	36	37	
III-IV	23	30	
Mobility, %			
Assisted	6	5	0.86
Independent	94	95	
Six-Minute Walking Distance, mts	294 ± 74	271 ± 92	0.14
5-time Sit-to Stand Test, sec	22.5 ± 5.1	23.9 ± 5.3	0.16

### Effect of the home-based training program on functional capacity and other parameters

At Baseline, 6MWD and 5STS time did not significantly differ between the two study groups (Table 1). At 6 months, both the 6MWD (*6 months*: 327 ± 86 m versus *baseline*: 294 ± 74 m; within-group comparison, *P* < 0.001) and the 5STS time (*6 months*: 19.8 ± 5.6 s versus *baseline*: 22.5 ± 5.1 s; within-group comparisons, *P* < 0.001) improved in the exercise group whereas they did not change in the control group (6MWD*, 6 months*: 270 ± 98 m versus *baseline:* 271 ± 92 m; within-group comparison, *P* = 0.98; 5STS, *6 months:* 23.1 ± 5.8 s versus 23.9 ± 5.3 s; within-group comparison, *P* = 0.25) (Table 2). The between-arms differences (6 months-baseline) in the 6MWD (+ 34.0 m, 95% CI: 14.4 to 53.5 m) and in the 5STS time changes (− 1.9 s, 95% CI: -3.6 to − 0.3 s) were both statistically significant (*P* = 0.001 and *P* = 0.024, respectively) (Table 2). The between-arms difference in 6MWD and 5STS time changes remained significant (*P* < 0.001 and *P* = 0.034, respectively) also after data adjustment for baseline variables which differed (with *P* < 0.10) between the two study arms as well as for baseline 6MWD and 5STS time.

**Table 2:** Within and between-arms differences in 6MWD and 5STS time. Data are mean and standard deviation or as mean and 95% CI

	Active group		Control group		Between-arms differences in changes (mean and 95% CI)
	*Baseline*	*6-month*	*Baseline*	*6-month*	
6MWD (m)	294 ± 74	327 ± 86*	271 ± 92	270 ± 98	+ 34.0 m (14.4 to 53.5 m)
5STS time (sec.)	22.5 ± 5.1	19.8 ± 5.6*	23.9 ± 5.3	23.1 ± 5.8	− 1.9 s (− 3.6 to − 0.3 s)

*P < 0.001 versus baseline

No significant effect of physical exercise was found on the incidence rate of all-cause [HR (active versus control arm): 0.68, 95% CI: 0.25–1.87, *P* = 0.46] and cardiovascular hospitalizations [HR: 1.18, 95% CI: 0.38–3.66, *P* = 0.78].

Changes in clinical and biochemical biomarkers occurring over the 6-month period are given in Additional file 1: Table S1.

### Quality of life

Within and between-arms differences of changes in KDQOL-SF components occurring over the 6-month period are detailed in Additional file 1: Table S2. As shown in Additional file 1: Table S2, cognitive function significantly reduced in patients of the control arm (*P* = 0.04) while it remained substantially unchanged in those of the active arm (P = 0.78) and the between-arms difference was statistically significant (*P* = 0.05). No other statistically significant differences were found between-groups differences in changes of KDQOL-SF components (Additional file 1: Table S2).

### Safety of the exercise program

No patient died during the trial. No angina episode or other major symptoms/complications during exercise were reported in the active arm of the trial. Symptoms of moderate intensity, not limiting the program execution, were reported in 54% of the session performed by 38 patients and included moderate fatigue (*n* = 31), “heavy legs” or leg pain (*n* = 29), moderate dyspnoea (*n* = 19), or other symptoms, including joint pain (*n* = 14). Six patients reported four symptoms, 12 reported three symptoms, 14 reported at least two symptoms, and 6 only reported one symptom during the exercise sessions. Overall, the training program was well tolerated and only 5 telephone calls were received by the rehabilitation team across the trial.

## Discussion

This secondary analysis of the EXCITE trial generates the hypothesis that a low intensity, home-based, personalized, physical exercise program improves physical performance and stabilizes cognitive function in dialysis patients aged > 65 years.

Dialysis patients have extremely low levels of physical functioning and exercise capacity, and are often physically inactive [15, 16]. NKF-KDOQI Guidelines formally recommend that all dialysis patients should be counselled and regularly encouraged by nephrology and dialysis staff to increase their level of physical activity and that physical functioning assessment and encouragement for participation in physical activity should be part of the routine patient care plan [4]. On the basis of this recommendation, we designed and performed a multicenter, randomized, controlled clinical trial, the EXerCise Introduction To Enhance performance in dialysis patients trial (EXCITE -NCT01255969), testing whether a personalized, home-based, low-intensity, physical exercise program improves the degree of fitness and quality of life in dialysis patients [5, 7, 17]. In the primary analysis of the EXCITE trial, i.e. in a dialysis population with a wide spectrum of age (from 25 to 91 years), we already documented that a home-based exercise program managed by dialysis staff improves physical performance and quality of life in dialysis patients [5]. In particular, over a 6-month time period we found that the 6MWD improved by 39 m in the exercise group and only by 3 m in the control group and the between groups difference was highly significant (*P* < 0.001). Similarly, the 5STS test time improved in the active arm (− 2.3 s) whereas it remained virtually unchanged in the control arm of the same trial (− 0.7 s) and again the between groups difference was statistically significant (*P* = 0.001). In the present analysis restricted to ESRD patients aged > 65 years, we found that in patients of the active arm the improvements in 6MWD (+ 33 m) and in 5STS test time (− 2.7 s) were of similar magnitude to those found in patients of the exercise arm of the primary analysis of the EXCITE trial (Δ 6MWD: + 39 m; Δ 5STS: -2.3 s) whereas no improvement in physical performance was observed in older adults patients of the control arm (Δ 6MWD: -1 m, Δ 5STS test time: − 0.8 s). These findings are of interest because for the first time we demonstrate that a low intensity, home-based, physical exercise program has an effect in older adults dialysis patients which is comparable to that observed in the general ESRD population with a wide age range (from 25 to 91 years).

The EXCITE trial also contemplated the assessment of quality of life in patients of the active and control arm over a 6-month time period. Cognitive function is an important component of quality of life and general health status which pertains attention, memory, language, perception, decision making, and problem solving [18–20]. The burden of cognitive impairment is higher in dialysis patients than in the general population and represents a disabling condition because it compromises quality of life, increases resource utilization, and results in suboptimal medical care [21–23]. In this secondary analysis of the EXCITE trial, we found that cognitive function significantly reduced in patients of the control arm (*P* = 0.04) while it remained substantially unchanged in those of the active arm (*P* = 0.78) and the between-arms difference was of borderline significance (*P* = 0.05). This result suggests that regular physical activity over a 6-month time period may stabilize the cognitive decline in older adults dialysis patients and generates the hypothesis that physical exercise may counterbalance the age-related worsening in cognitive function in the dialysis population. However, this latter hypothesis demands to be specifically investigated in an appropriately designed randomized clinical trial.

The effect of physical exercise on clinical outcomes in dialysis patients represents a tantalizing research area and various exercise programmes have been proposed. Koh et al., in a pilot study in older dialysis patients [24], found no significant differences between intradialytic or home-based exercise training and usual care for physical function as assessed by 6MWD. The results emerged in our study do not contrast with those of Koh et al. In the Koh’s study, over a 6 month period, the increase in 6MWD in patients of the home-based exercise arm was more than double than that found in patients of the usual care arm (11% versus 5%) and the lack of a significant difference between the magnitude of these effects most likely depends on the extremely low sample size of the Koh’s study (from an initial population of 70 patients, the 6 month data were only available in 15 patients of the home-based exercise arm and in 16 patients of the usual care arm).

Another novel finding of our study is that the physical exercise program is safe and well tolerated in older adults ESRD patients. Moderate intensity symptoms such as moderate fatigue, leg pain, dyspnea and joint pain were recorded in 54% of session performed by 38 patients and only 5 phone calls for problems related with exercise tolerance were received by the rehabilitation team that supervised the training of patients and the safety of the intervention. Furthermore, no episodes of angina or other major symptoms have been reported in either of the two analyzed groups.

Major limitations of our study are that it is a post-hoc analysis of a randomized clinical trial which did not aim to examine a geriatric population and the fact that the number of patients considered here is about one half of that of the original trial (115 versus 227). Thus, our findings need to be interpreted very cautiously and formally confirmed in a randomized clinical trial specifically enrolling older adult patients on dialysis. Another limitation of our study is that the protocol did not contemplate frailty assessment, dietary change or dietary diary and other physical assessment.

## Conclusions

In conclusion, this secondary analysis of the EXCITE trial generates the hypothesis that a simple, home-based, personalized exercise program is well tolerated and improves physical performance and stabilizes cognitive function in older adults dialysis patients. Our trial represents a stimulus to nephrologists for beginning long term trials testing whether a simple exercise training could reduce the risk of many adverse health conditions and increase life expectancy and quality of life in a very high-risk population such as older adults patients on chronic dialysis.

## Additional file

Additional file 1: **Table S1.** Within and between arms differences in hemodynamic and biochemical data. **Table S2.** Within and between arms differences in KDQOL-SF components (DOC 78 kb)

## Abbreviations

5STS: 5 Sit-To-Stand
6MWD: 6 Minute Walking Distance
BMI: Body Mass Index
BP: Blood Pressure
CKD: Chronic Kidney Disease
ESRD: End-Stage Renal Disease
EXCITE: EXerCise Introduction To Enhance
ITT: Intention To Treat
KDQOL-SF: Kidney Disease Quality of Life- Short Form
NKF-KDOQI: National Kidney Foundation-Kidney Disease Outcomes Quality Initiative
PTH: Parathyroid Hormone
QoL: Quality of Life
SD: Standard Deviation
TIA: Transient Ischemic Attack
