# Supplementary material for: Effect of a home based, low intensity, physical exercise program in older adults dialysis patients: a secondary analysis of the EXCITE trial
Source: BMC Geriatr. 2018 Oct 20;18:248. doi: 10.1186/s12877-018-0938-5 (PMC6196029; doi:10.1186/s12877-018-0938-5)
Supplement: Supplementary file 1 — Table S1. Within and between arms differences in hemodynamic and biochemical data. Table S2. Within and between arms differences in KDQOL-SF components (DOC 78 kb) [file 12877_2018_938_MOESM1_ESM.doc]

**Supplementary Table 1.** Within and between arms differences in hemodynamic and biochemical data.

|  | **Active group** | | | | **Control group** | | | | **Active vs Control group** | |
| --- | --- | --- | --- | --- | --- | --- | --- | --- | --- | --- |
|  | ***baseline*** | ***6 months*** | ***Changes***  ***(6 months –baseline)*** | **P** | ***baseline*** | ***6 months*** | ***Changes***  ***(6 months-baseline)*** | **P** | ***Between-arms***  ***difference in changes*** | **P** |
| Systolic BP, mmHg | ***134 (128-139) | 135 (131-139) | 1.4  (from -3.1 to 6.0) | 0.53 | 125 (121-129) | 129 (124-133) | 4.1  (from -0.6 to 8.8) | 0.09 | 2.7  (from -3.9 to 9.2) | 0.42 |
| Diastolic BP, mmHg | 70 (68-72) | 70 (68-73) | 0.5  (from -1.8 to 2.8) | 0.66 | 66 (63-69) | 67 (64-70) | 0.7  (from -1.9 to 3.3) | 0.58 | 0.2  (-3.2 to 3.7) | 0.90 |
| Heart rate, beats/min | 72 (70-75) | 73 (71-76) | 1.1  (from -1.3 to 3.5) | 0.36 | 73 (71-75) | 72 (70-75) | -0.4  (from -2.3 to 1.6) | 0.71 | -1.5  (from -4.5 to 1.6) | 0.35 |
| Albumin, g/dl | *3.8 (3.7-3.9) | 3.6 (3.5-3.8) | -0.14  (from -0.24 to -0.03) | 0.01 | 3.6 (3.5-3.8) | 3.6 (3.5-3.7) | -0.06  (from -0.16 to 0.05) | 0.29 | 0.08  (from -0.06 to 0.23) | 0.27 |
| Cholesterol, mg/dl | 161(150-172) | 164 (153-174) | 2.38  (from - 3.89 to 8.66) | 0.45 | 163 (153-174) | 169 (158-179) | 5.19  (from -2.23 to 12.62) | 0.17 | 2.81  (from -6.78 to 12.40) | 0.56 |
| Triglycerides, mg/dl | 155 (133-178) | 168 (138-198) | 12.49  (from -7.42 to 32.39) | 0.21 | 143 (124-162) | 160 (135-186) | 17.55  (from 1.76 to 33.34) | 0.03 | 5.07  (from -19.99 to 30.12) | 0.69 |
| Glycemia, mg/dl | 112 (99-123) | 116 (97-136) | 5.0  (from -13.9 to 23.9) | 0.59 | 104 (96-113) | 103 (95-110) | -1.8  (from -7.9 to 4.2) | 0.54 | -6.9  (from -26.6 to 12.9) | 0.49 |
| Hemoglobin, g/dl | **11.6 (11.3-12.0) | 11.8 (11.4-12.2) | 0.18  (from -0.25 to 0.61) | 0.40 | 11.0 (10.7-11.4) | 11.4 (11.1-11.7) | 0.39  (from -0.03 to 0.82) | 0.07 | 0.21  (from -0.38 to 0.81) | 0.48 |
| Calcium, mg/dl | 8.9 (8.6-9.1) | 8.6 (8.3-8.9) | -0.22  (from -0.55 to 0.11) | 0.18 | 8.9 (8.7-9.1) | 8.5 (8.1-8.9) | -0.39  (from -0.82 to 0.05) | 0.80 | -0.17  (from -0.70 to 0.37) | 0.54 |
| Phosphate, mg/dl | 4.7 (4.2-5.1) | 4.7 (4.4-5.1) | 0.08  (from -0.38 to 0.54) | 0.73 | 4.1 (3.8-4.4) | 4.2 (3.9-4.5) | 0.09  (from -0.25 to 0.43) | 0.59 | 0.01  (from -0.55 to 0.58) | 0.97 |
| PTHi, pg/ml | 347 (265-491) | 75 (67-82) | 39.13  (from -57.49 to 135.77) | 0.42 | 267 (222-312) | 255 (196-315) | -11.49  (from -54.34 to 31.34) | 0.59 | -50.63  (from -155.66 to 54.40) | 0.34 |
| CRP, mg/L | 7.4 (5.3-9.5) | 12.1 (6.9-17.2) | 4.70  (from -1.02 to 10.42) | 0.10 | 7.6 (5.6-9.6) | 9.3 (5.1-13.6) | 1.74  (from -2.10 to 5.58) | 0.37 | -2.97  (from -9.78 to 3.84) | 0.39 |

**Data are given as mean and 95% CI.**

* P=0.03 versus baseline albumin value of the control group.

**P=0.02 versus baseline Hemoglobin value of the control group.

***P=0.01 versus baseline systolic BP value of the control group.

**BP**: blood pressure; **PTHi**: intact parathyroid hormone; **CRP**: c-reactive protein

**Supplementary Table 2.** Within and between arms differences in KDQOL-SF components.

|  | **Active group** | | | | **Control group** | | | | **Active vs Control group** | |
| --- | --- | --- | --- | --- | --- | --- | --- | --- | --- | --- |
|  | ***baseline*** | ***6 months*** | ***6 months-baseline*** | **P** | ***baseline*** | ***6 months*** | ***6 months-baseline*** | **P** | ***Between-arms***  ***difference in changes*** | **P** |
| **Symptom/problem list** | 75.1 (69.4-80.8) | 74.2 (69.1-79.3) | -0.9  (from -5.5 to 3.6) | 0.68 | 70.8 (66.1-75.5) | 69.8 (64.9-74.7) | -1.0  (from -6.2 to 4.2) | 0.70 | -0.1  (from -7.2 to 7.0) | 0.98 |
| **Effects of Kidney Disease** | 64.4 (56.4-72.4) | 62.8 (55.7-69.9) | -1.6  (from -9.5 to 6.3) | 0.68 | 63.0 (56.9-69.1) | 61.8 (56.3-67.3) | -1.2  (from -7.7 to 5.3) | 0.70 | 0.4  (from -9.6 to 10.3) | 0.94 |
| **Burden of Kidney Disease** | 41.2 (31.8-50.6) | 41.0 (33.6-48.4) | -0.2  (from -8.0 to 7.6) | 0.96 | 38.5 (31.1-45.9) | 39.8 (31.0-48.6) | 1.4  (from -7.7 to 10.5) | 0.76 | 1.5  (from -10.4 to 13.5) | 0.79 |
| **Work status** | 13.4 (3.2-23.6) | 13.2 (4.0-22.4) | -0.2  (from -6.6 to 6.1) | 0.94 | 9.3 (2.2-16.4) | 11.3 (3.5-19.1) | 2.0  (from -6.7 to 10.7) | 0.65 | 2.2  (from -8.7 to 13.1) | 0.68 |
| **Cognitive function** | 64.1 (56.1-72.1) | 65.0 (57.0-73.0) | 0.8  (from -4.9 to 6.5) | 0.78 | 67.4 (60.3-74.5) | 57.8 (49.2-66.4) | -9.6  (from -18.5 to -0.7) | 0.04 | -10.4  (from -21.6 to 0.8) | **0.05** |
| **Quality of social interaction** | 78.3 (73.2-83.4) | 77.3 (72.4-82.2) | -0.98  (from -5.7 to 3.8) | 0.68 | 81.4 (75.5-87.3) | 74.3 (67.8-80.8) | -7.1  (from -13.7 to -0.5) | 0.04 | -6.1  (from -14.6 to 2.4) | 0.13 |
| **Sexual function** | 77.2 (64.9-89.5) | 69.7 (55.0-84.4) | -7.5  (from -21.4 to 6.4) | 0.28 | 61.2 (45.7-76.7) | 63.0 (48.1-77.9) | 1.7  (from -16.1 to 19.5) | 0.84 | 9.2  (from -12.6 to 31.1) | 0.40 |
| **Sleep** | 60.5 (54.4-66.6) | 64.4 (58.5-70.3) | 3.9  (from -0.5 to 8.4) | 0.08 | 54.8 (49.3-60.3) | 54.9 (49.8-60.0) | 0.2  (from -5.38 to 6.2) | 0.95 | -3.7  (from -11.6 to 4.2) | 0.32 |
| **Social support** | 72.8 (65.7-79.9) | 68.6 (61.2-76.0) | -4.2  (from -11.3 to 2.9) | 0.23 | 69.9 (63.6-76.2) | 67.7 (61.4-74.0) | -2.2  (from -9.8 to 2.6) | 0.56 | 2.0  (from -8.4 to 12.4) | 0.70 |
| **Dialysis staff encouragement** | 72.2 (67.7-76.7) | 73.5 (68.4-78.6) | 1.3  (from -3.5 to 6.1) | 0.59 | 76.6 (72.7-80.5) | 74.2 (70.3-78.1) | -2.3  (from -7.3 to 2.6) | 0.35 | -3.6  (from -10.6 to 3.3) | 0.29 |
| **Patients satisfaction** | 76.7 (68.9-84.5) | 74.7 (67.3-82.1) | -1.9  (from -7.5 to 3.6) | 0.48 | 74.2 (67.7-80.7) | 70.1 (63.6-76.6) | -4.1  (from -12.2 to 4.1) | 0.32 | -2.1  (from -12.4 to 8.2) | 0.67 |
| **Physical functioning** | 48.6 (38.0-59.2) | 50.0 (39.6-60.4) | 1.4  (from -5.7 to 8.5) | 0.69 | 45.2 (37.6-52.8) | 41.4 (33.6-49.2) | -3.8  (from -12.0 to 4.4) | 0.36 | -5.2  (from -16.2 to 5.8) | 0.34 |
| **Role physical** | 52.3 (37.4-67.2) | 35.1 (20.2-50.0) | -17.2  (from -32.4 to -1.9) | 0.03 | 29.7 (17.4-42) | 15.8 (6.6-25.0) | -13.8  (from -28.3 to 0.7) | 0.06 | 3.4  (from -17.5 to 24.2) | 0.75 |
| **Pain** | 54.8 (44.2-65.4) | 59.5 (50.3-68.7) | 4.7  (from -4.6 to 14.0) | 0.31 | 56.0 (47.0-65.0) | 51.6 (42.6-60.6) | -4.4  (from -16.3 to 7.5) | 0.46 | -9.1  (from -24.4 to 6.3) | 0.23 |
| **General health** | 37.1 (30.6-43.6) | 35.5 (30.0-41.0) | -1.5  (from -8.0 to 4.9) | 0.63 | 34.1 (29.0-39.2) | 29.6 (24.5-34.7) | -4.4  (from -10.3 to 1.4) | 0.13 | -2.9  (from -11.6 to 5.7) | 0.50 |
| **Emotional well - being** | 57.7 (50.1-65.3) | 59.3 (52.0-66.6) | 1.5  (from -4.7 to 7.8) | 0.62 | 61.2 (54.3-68.1) | 57.1 (50.4-63.8) | -4.1  (from -11.7 to 3.4) | 0.27 | -5.7  (from -15.8 to 4.4) | 0.24 |
| **Role emotional** | 63.6 (48.7-78.5) | 53.5 (37.6-69.4) | -10.1  (from -24.1 to 3.9) | 0.15 | 45.0 (30.9-59.1) | 29.2 (18.2-40.2) | -15.8  (from -33.2 to 1.6) | 0.07 | -5.7  (from -28.4 to 16.9) | 0.60 |
| **Social function** | 87.1 (79.8-94.4) | 78.3 (69.7-86.9) | -8.8  (from -17.0 to -0.6) | 0.04 | 84.1 (78.2-90.0) | 80.7 (73.4-88.0) | -3.4  (from -9.7 to 2.9) | 0.28 | 5.4  (from -4.6 to 15.4) | 0.29 |
| **Energy/fatigue** | 46.5 (38.7-54.3) | 41.2 (32.8-49.6) | -5.3  (from -12.6 to 2.0) | 0.15 | 45.8 (38.9-52.7) | 40.8 (33.7-47.9) | -5.0  (from -12.9 to 3.0) | 0.22 | 0.34  (from -10.7 to 11.3) | 0.95 |

**Data are mean and 95% CI.**
